# Belief about Nicotine Modulates Subjective Craving and Insula Activity in Deprived Smokers

**DOI:** 10.3389/fpsyt.2016.00126

**Published:** 2016-07-13

**Authors:** Xiaosi Gu, Terry Lohrenz, Ramiro Salas, Philip R. Baldwin, Alireza Soltani, Ulrich Kirk, Paul M. Cinciripini, P. Read Montague

**Affiliations:** ^1^Wellcome Trust Centre for Neuroimaging, University College London, London, UK; ^2^School of Behavioral and Brain Sciences, The University of Texas at Dallas, Dallas, TX, USA; ^3^Human Neuroimaging Laboratory, Virginia Tech Carilion Research Institute, Roanoke, VA, USA; ^4^Menninger Department of Psychiatry and Behavioral Sciences, Baylor College of Medicine, Houston, TX, USA; ^5^Department of Psychological and Brain Sciences, Dartmouth College, Hanover, NH, USA; ^6^Institute of Psychology, University of Southern Denmark, Odense, Denmark; ^7^Department of Behavioral Science, The University of Texas MD Anderson Cancer Center, Houston, TX, United States; ^8^Department of Physics, Virginia Polytechnic Institute, State University, Blacksburg, VA, USA

**Keywords:** nicotine addiction, belief, craving, interoception, insula, fMRI

## Abstract

Little is known about the specific neural mechanisms through which cognitive factors influence craving and associated brain responses, despite the initial success of cognitive therapies in treating drug addiction. In this study, we investigated how cognitive factors such as beliefs influence subjective craving and neural activities in nicotine-addicted individuals using model-based functional magnetic resonance imaging (fMRI) and neuropharmacology. Deprived smokers (*N* = 24) participated in a two-by-two balanced placebo design, which crossed beliefs about nicotine (told “nicotine” vs. told “no nicotine”) with the nicotine content in a cigarette (nicotine vs. placebo) which participants smoked immediately before performing a fMRI task involving reward learning. Subjects’ reported craving was measured both before smoking and after the fMRI session. We found that first, in the presence of nicotine, smokers demonstrated significantly reduced craving after smoking when told “nicotine in cigarette” but showed no change in craving when told “no nicotine.” Second, neural activity in the insular cortex related to craving was only significant when smokers were told “nicotine” but not when told “no nicotine.” Both effects were absent in the placebo condition. Third, insula activation related to computational learning signals was modulated by belief about nicotine regardless of nicotine’s presence. These results suggest that belief about nicotine has a strong impact on subjective craving and insula responses related to both craving and learning in deprived smokers, providing insights into the complex nature of belief–drug interactions.

## Introduction

Craving is a core symptom of drug addiction (1–3) and has been proven to be much more difficult to treat than physical dependency symptoms (4). Although certain mechanisms of drug addiction such as the malfunctioning dopaminergic system have been relatively well characterized (5–7), it has become clear that complex interactions exist between drug beliefs (expectancies) and pharmacological effects, and that these interactions may impose a profound influence on treatment outcome (8, 9). Previous work has shown that drug beliefs can modify neural activity related to alcohol (10–12), nicotine (13–15), and cocaine (16, 17). In particular, our recent work (15) examined the impact of beliefs on nicotine-induced learning signals in a group of non-deprived smokers using functional magnetic resonance imaging (fMRI) and a reward learning task. We found that in non-deprived smokers, belief about nicotine’s presence modulated both neural learning signals in the ventral striatum as well as learning behavior when participants smoked a cigarette with nicotine (15). These results demonstrate that belief has a powerful effect in overriding the effects of nicotine on reward learning. It remained unclear, however, whether such belief–drug interactions could modulate other aspects of drug addiction such as craving. The current study uses a balanced placebo design to directly examine the impact of belief about drugs (i.e., nicotine) on subjective craving and associated patterns of neural activation measured by fMRI among deprived smokers.

The insular cortex is one of the key brain areas most consistently implicated in drug addiction (1–3, 18–21). Insular lesions lead to abstinence from smoking in humans (19) and reduced craving and drug-seeking behavior in rats (22, 23). Activation of the insula has been associated with exposure to smoking cues (24). Different from the ventral striatum, a dopaminergic region responsible for reinforcement learning and motivational abnormalities in addiction (15, 25), the insula has been typically considered to encode the interoceptive effects of drug taking, craving, and urges (2, 26). While craving and reinforcement learning have mostly been considered separately in previous studies, converging evidence suggests that interoception has a substantial influence on learning and other cognitive processes (27–29), and that the insula is involved in a much wider range of cognitive functions than traditionally thought. Using learning and decision-making paradigms, recent studies have shown that anterior insula activation correlates with value prediction and prediction errors (30), risk and risk prediction errors (31), and subjective feelings during decision-making (32). Mid insula has been reported to integrate homeostatic with cognitive information (33). These findings lead to the recent proposal that insula is a critical hub for linking bodily information with decision-making signals (15, 29). It is therefore important to investigate how the insula responses related to both craving and reinforcement learning can be modulated by beliefs in deprived smokers while under the influence of nicotine.

**Figure 1:**
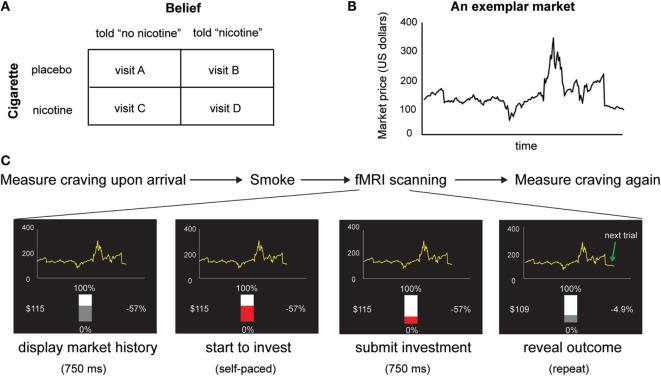
**(A)** Experimental design. We used a within-subject design with two factors: belief (told “no nicotine” vs. told “nicotine”) and cigarette (placebo vs. nicotine). **(B)** An exemplar stock market used in the task. Participants make 20 sequential decisions in each market and there are 10 markets in total. **(C)** Time course of the experiment. In each visit, subjective craving was measured upon arrival, before smokers were given a cigarette with or without nicotine to smoke and were told that the cigarette had nicotine or had no nicotine. Immediately after smoking, participants performed a sequential choice task. Craving was measured again immediately after the fMRI session.

Based on previous finding of the effect of belief–drug interaction on learning in non-deprived smokers, we hypothesized that beliefs about nicotine would also modulate nicotine-induced subjective craving and insula responses in deprived smokers whose craving levels are elevated. To test this hypothesis, we employed a within-subject, balanced placebo design (Figure 1A) with two experimental factors: belief (told “nicotine,” told “no nicotine”) and cigarette (nicotine, placebo/de-nicotinized), together with fMRI and a reward learning task (Figures 1B,C) in 24 chronic smokers. Similar to our previous study (15), smokers were given a cigarette with nicotine or without nicotine and were told that the cigarette either contained nicotine or not, in four separate visits. In contrast to our previous study (15), smokers stayed abstinent from smoking starting from the midnight before the experiment. We measured craving using self-reports both before participants smoked a cigarette and after the fMRI session. We examined the impact of beliefs on changes in craving (i.e., post vs. pre-smoking). We also measured neural activity indexed by blood oxygen level dependent (BOLD) when subjects performed a sequential financial investment task. During each trial of the investment task, the subject places a bet *b*_t_, observes a fractional change in market price change *r*_t_ = (*p*_t_–*p*_t −1_)/*p*_t −1_, where *p*_t_ is the market price at time *t*, and receives a gain or loss *g*_t_. This fractional market return *r*_t_ is an important value signal known to guide learning and is associated with neural responses in mesolimbic dopaminergic regions (15). Here, we used the *choice-independent* and *passive* value signal *r*_t_, instead of the actual gain/loss (*g*_t_ = *r*_t_*b*_t_), or the prediction error, as our main learning signal to avoid risk preference – induced variability in smokers’ bets as a potential confounding. Our hypothesis yields two main predictions. First, based on previous findings on the effects of beliefs (15, 34, 35), we predicted that belief about nicotine would modulate reported change in craving levels induced by nicotine intake in deprived smokers. Second, we predicted that belief would also modulate neural activity changes in brain regions integrating interoceptive information with learning signals such as the insular cortex.

## Materials and Methods

### Participants

We recruited 28 chronic smokers from the community population in Houston, TX, USA, who were not interested in quitting smoking. Four smokers were excluded because they had carbon monoxide (CO) levels outside of acceptable range [>30 parts per million (ppm), based on the mean exhaled CO level of non-deprived heavy smokers provided by manufacturer^1^]. Other exclusion criteria include: (1) left handedness; (2) claustrophobia; (3) DSM-IV Axis I or II diagnosis (36), exclusive of nicotine dependence; (4) pregnancy; (5) contraindications to MRI: pacemaker, aneurysm clips, neurostimulators, cochlear implants, metal in eyes, steel worker, or other implants; (6) active medical or neurologic disorder; (7) history of drug dependence (other than tobacco or alcohol); (8) history of head injuries resulting in loss of consciousness >10 min; (9) non-smoker. Because about 80% of tobacco users are also alcohol users in the general population (37), we included smokers regardless of their history of alcohol use. This yielded a final sample of 24 smokers. The demographics and smoking history of the smokers are as follows (mean ± SD): age, 41 ± 10 years; education, 15 ± 2.6 years; smoking history, 25 ± 11.5 years; daily cigarettes, 15 ± 7. Participants had normal or adjusted to normal vision and were informed of the study requirements and provided written consent prior to participation. The study was approved by the Institutional Review Board of Baylor College of Medicine.

### Experimental Procedure, Stimuli, and Task

We used a within-subject, placebo-balanced design that is similar to the one used in our previous study (15). All smokers were instructed to stop smoking beginning at midnight prior to the experiment day. Deprivation status was confirmed by exhaled CO levels measured upon smokers’ arrival at the laboratory. The average CO level of smokers was 10.4 ± 5.7 ppm, which is significantly lower than average CO level in non-deprived smokers [e.g., compared to mean = 28.5 ppm in a non-deprived smoker group (15)].

Each smoker came to the laboratory four times on four separate days and participated in one of the following conditions during each visit (Figure 1A): told “nicotine” and received a cigarette with nicotine (Quest Brand, 0.6 milligrams of nicotine), told “nicotine” and received a de-nicotinized cigarette (Quest Brand, 0.06 milligrams of nicotine), told “no nicotine” and received a cigarette with nicotine, and told “no nicotine” and received a de-nicotinized cigarette. The 0.06 mg Quest cigarette has very low nicotine content; while this is not “zero” nicotine, previous studies suggest that cigarettes with this level of nicotine are considered by smokers to be subjectively less reinforcing than normal nicotine content cigarettes (38, 39).

The experiment was designed to be double blind. Before smoking, participants were told that they would receive either a nicotine cigarette or a de-nicotinized cigarette and that the experimenter would inform them about the nicotine content. Participants remained blind to the actual nicotine content of the cigarette during the experiment, and were only debriefed after they completed all four sessions. Although the experimenters giving the cigarette to the participant developed their own beliefs of the nicotine content of the cigarette for some sessions, the double-blind protocol was not broken during data collection. Two other researchers who were completely blind to data collection performed data analysis.

The order of the four visits was randomly assigned to each subject. Upon arrival at the laboratory, participants completed the Shiffman-Javik Withdrawal Questionnaire (SJWQ) (40) for craving, the Positive and Negative Affect Schedule (PANAS) (41) for mood and affect, and MRI safety-related questions. All participants then received a cigarette to smoke immediately before the scanning session. SJWQ and PANAS were administered again after the scanning session.

Participants performed a reward learning task (Figures 1B,C) in which they made 20 sequential investment decisions for each of the 10 markets, yielding 200 trials per fMRI session and a total of four sessions. The markets were taken from real historic markets (15, 42–44). Different markets were used for each visit to prevent memory effects. At the beginning of each fMRI session, participants were endowed with 100 monetary units and were informed that their final payment would be scaled according to their earning in the task. For each market, the subject used a two-button box to move a slide bar to make 20 sequential investment decisions *b_t_* (0~100% of current portfolio) without a time constraint. Seven hundred fifty milliseconds after they submitted their choices, the market price *p_t_* was revealed and the fractional market price change and participants’ portfolio were updated. Market information for all previous segments then remained on the screen. The slide bar then changed from gray to red after another 750 ms, and participants started to make investment decisions for the next market segment. Different markets were used for the four visits.

### Behavioral Data Analysis Using a Linear Mixed-Effects Multiple Regression Model

We used a linear mixed-effects multiple regression model (45) to examine the impact of the value signal *r_t_* on subjects’ next bet *b*_t+1_, similar to the methods described in our previous work (46). The value signal at time *t r_t_*, is defined as the relative change in market price (*p_t_* − *p_t_*_ − 1_)/*p_t_*_− 1._ The regression was performed simultaneously across all four conditions by coding the four conditions (told “no nicotine” and received placebo, told “nicotine” and received placebo, told “no nicotine” and received nicotine, told “nicotine” and received nicotine) as four indicator variables (told0-nic0, told1-nic0, told0-nic1, told1-nic1) and by including a term in the regression of the form CONDITION regressor for each indicator and regressor (see Table S1 in Supplementary Material for a complete list of regressors and fixed-effect coefficients). Specifically, if we let *l*_T0,*t*_ be the indicator function for trials *t* where the subject has been told the cigarette does not contain nicotine, *l*_T1,*t*_ be the indicator function for trials *t* where the subject has been told the cigarette contains nicotine, *l*_N0,*t*_ be the indicator function for trials *t* where the subject receives a placebo cigarette, and *l*_N1,*t*_ be the indicator function for trials *t* where the subject receives a cigarette with nicotine, then the model for subject *j* is given by:

$$\begin{array}{l} {\tilde{b}}_{t+1,j}=\beta_{1}\;\cdot 1_{T0,t}\;\cdot 1_{N0,t}\;+\beta_{2}\;\cdot 1_{T1,t}\;\cdot 1_{N0,t}\;+\beta_{3}\;\cdot 1_{T0.t}\;\cdot 1_{N1,t}\;+\beta_{4}\;\cdot 1_{T1,t}\;\cdot 1_{N1,t}+\\ \;(\beta_{5}\;\cdot \;1_{T0,t}\;\cdot \;{\text{1}}_{N0,t}\;+\beta_{6}\;\cdot \;{\text{1}}_{T1,t}\;\cdot \;1_{N0,t}\;+\beta_{7}\;\cdot \;1_{T0,t}\;\cdot \;1_{N1,t}\;+\beta_{8}\;\cdot \;1_{T1,t}\;\cdot \;1_{N1,t})\cdot {\tilde{b}}_{t,j}+\\ \;(\beta_{9}\;\cdot 1_{T0,t}\;\cdot 1_{N0,t}\;+\beta_{10}\;\cdot 1_{T1,t}\;\cdot 1_{N0,t}\;+\beta_{11}\;\cdot 1_{T0,t}\;\cdot 1_{N1,t}\;+\beta_{12}\;\cdot 1_{T1,t}\;\cdot 1_{N1,t})\cdot r_{t,j}+\text{ (}Z_{j}\cdot u_{j}{)}_{t}+\varepsilon_{t}, \end{array}$$

where the random effect for subject *j* is given by:

(Zj  uj)t= (u1,j 1T0,t 1N0,t +u2,j 1T1,t 1N0,t +u3,j 1T0,t 1N1,t+ u4,j 1T1,t 1N1,t)+(u5,j 1T0,t 1N0,t +u6,j1T1,t1N0,t+u7,j1T0,t1N1,t+u8,j1T1,t1N1,t)b˜t +(u9,j1T0,t1N0,t+u10,j1T1,t1N0,t+u11,j1T0,t1N1,t+u12,j1T1,t1N1,t)rt

here *Z_j_* is the design matrix for the random effects, *u*_j_ is the vector of random effects for subject *j*, ${\tilde{b}}_{\text{t,}j}$the within-subject *z*-normalized (over the entire experiment for subject *j*) bet, ϵ_t,*j*_~*N*(0,σ^2^)IID, *u*_j_~*N*(0,∑), IID, and ϵ and *u* are independent. Linear contrasts were then carried out to test the significance of differences between coefficients. The analyses were carried out in R(Team) (47) with the function *lme* (for the mixed regression) in package *lmer* (48) and function *estimable* (for the linear contrasts) in package *gmodels* (49). Statistical significance was determined at *P* < 0.05 two tailed. The first and last bet of each market was excluded to keep consistent with the fMRI analysis.

### Image Acquisition and fMRI Data Preprocessing

High-resolution T1-weighted scans (1.0 mm × 1.0 mm × 1.0 mm) were acquired using a Magnetization-Prepared Rapid Acquisition with Gradient Echo (MP-RAGE) sequence. Functional images were acquired using echo-planar imaging (EPI), and angled 30° with respect to the anterior–posterior commissural line. The detailed settings for the functional imaging were: repetition time (TR) = 2000 ms; echo time (TE) = 25 ms; flip angle = 90°; 37 slices; voxel size: 3.4 mm × 3.4 mm × 4.0 mm. The functional scans were realigned to the first volume (the mean functional image), coregistered to the T1 image, normalized to a standard template (MNI, Montreal Neurological Institute), and spatially smoothed with an 8 mm × 8 mm × 8 mm full-width-at-half-maximum (FWHM) Gaussian kernel using statistical parametric mapping (SPM8; Wellcome Department of Imaging Neuroscience, London, UK^2^).

### General Linear Modeling of fMRI Data

The anatomical and functional imaging was conducted on a 3.0-T Siemens Trio scanner at Baylor College of Medicine in Houston, TX, USA. Event-related analyses of the fMRI data were conducted using statistical parametric mapping (SPM8; Wellcome Department of Imaging Neuroscience, London, UK) (see text footnote 2). GLM was conducted for the functional scans from each participant to identify the relationship between task events and hemodynamic responses. Regressors related to visual and motor events were created by convolving a train of delta functions representing the sequence of individual events with the default SPM basis function, which consists of a synthetic hemodynamic response function composed of two gamma functions. The following visual and motor events were included in the GLM: market type screen; initial market history screen; key press; first market price reveal; market price reveal round 2–19; last market price reveal. Six parameters generated during motion correction were entered as covariates. *r_t_* was entered as parametric regressor at the onset of “market price reveal round 2–19,” Regressors were orthogonalized in the standard SPM8 fashion.

For craving-related activations, post-smoking craving scores were entered as covariates for parameter estimates of “market price reveal round 2–19” in a second-level factorial analysis with the following factors: belief (told “no nicotine” vs. told “nicotine”) and cigarette (placebo vs. nicotine). For activations related to both craving and market value *r_t_*, post-smoking craving scores were entered as covariates for parameter estimates of *r_t_* in a second-level factorial analysis with the same two factors. Significant activations were identified at the voxel level of *P* < 0.05 corrected for family wise errors (*P_FWE_* < 0.05) for single conditions, and voxel level of *P* < 0.05 uncorrected in conjunction with a cluster threshold of *k* > 54 to correct for multiple comparison at *P* < 0.05 at the cluster level for contrasts between conditions based on a Monte Carlo simulation (50, 51).

Independent regions of interest (ROIs) were created using the MarsBaR toolbox (http://marsbar.sourceforge.net/) with a sphere of 5-mm radius (640 mm^3^, 10 voxels) centered at left anterior insula [−38 18 −2], left mid insula [−38 2 4], left posterior insula [−38 −14 4], right anterior insula [36 22 4], right mid insula [40 6 4], right posterior insula [40 −14 4], based on the search term “interoceptive” entered into a meta-analysis in Neurosynth^3^; left ventral striatum [−12 8 −6], right ventral striatum [12 10 −6], based on value-based activation from a previous study using a similar task (44).

## Results

### Effect of Belief on Learning Behavior

There was no main effect of belief or nicotine on learning behavior (see Table S1 in Supplementary Material for results based on learning behavior using a linear mixed-effects multiple regression model). Average reaction times (RT) of the four conditions (told “no nicotine” and smoked placebo, told “nicotine” and smoked placebo, told “no nicotine” and smoked nicotine, told “nicotine” and smoked nicotine) were 2.93 ± 1.10 s, 2.78 ± 0.81 s, 2.88 ± 1.01 s, 2.92 ± 0.95 s respectively. Average task lengths of the four conditions (told “no nicotine” and smoked placebo, told “nicotine” and smoked placebo, told “no nicotine” and smoked nicotine, told “nicotine” and smoked nicotine) were 878 ± 219 s, 849 ± 162 s, 868 ± 201 s, 878 ± 190 s, respectively. There was no main effect of belief or nicotine on RT or task length (*P*s > 0.1).

### Impact of Belief on Subjective Craving

**Figure 2:**
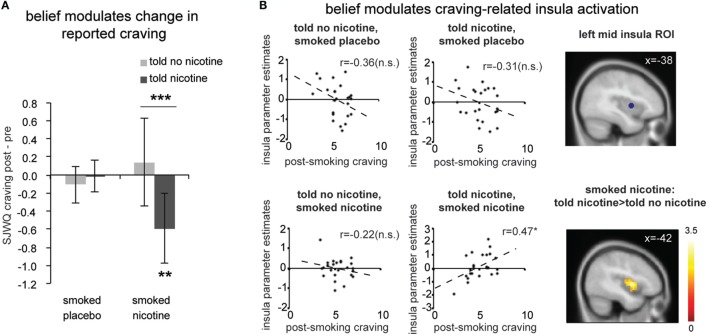
**(A)** Change in subjective craving measured by the Shiffman-Javik withdrawal questionnaire (SJWQ). There was a significant interaction between belief and nicotine (*P* < 0.05) and no significant main effect for belief or nicotine (*P*s > 0.05). **(B)** Both region-of-interest (ROI, *P* < 0.05) and whole brain analyses (*P* < 0.05 corrected) suggests that post-smoking craving significantly correlated with left mid insula activation only when smokers were told “nicotine’ and smoked nicotine, but not in other conditions. *P ≤ 0.05, ***P ≤ 0.005. n.s., not significant. Activation maps were displayed at *P* < 0.05 for visualization purpose. Error bar represents 95% confidence interval.

We calculated change in craving, that is, the difference between the two craving measurements (Δcraving = post-smoking/scanning craving – pre-smoking/scanning craving); positive scores indicate increased craving and negative scores indicate decreased craving after the experimental manipulation. There was a significant interaction between belief (what one was told) and nicotine (what one received) [Figure 2A; *F*_(1,23)_ = 7.66, *P* < 0.05] and no significant main effect for belief [*F*_(1,23)_ = 4.08, *P* > 0.05] or nicotine [*F*_(1,23)_ = 1.14, *P* > 0.2]. *Post hoc* comparisons indicated that belief-modulated change in craving when deprived smokers smoked a cigarette with nicotine [paired *t*-test *t*_(23)_ = 3.24, *P* < 0.005]. More specifically, smokers reported a significant reduction in craving when they smoked a nicotine cigarette and were told “nicotine” [one-sample *t*_(23)_ = −3.02, *P* < 0.01]; such decrease in craving was absent in the case of being told “no nicotine” and smoked a nicotine cigarette (*P* > 0.1). This effect of belief was not observed when participants smoked a de-nicotinized cigarette [*t*_(23)_ = −0.52, *P* > 0.6].

We further examined the effect of belief on the time lag between the two measurements and general mood measured by PANAS to rule out the possibility that the effect of belief on craving was due to differences in the time lag between two measurements or changes in general mood. There was no difference in time lag between any conditions (Figure S1A in Supplementary Material; *P*s > 0.1). Neither belief nor nicotine had an impact on changes in positive mood (Figure S1B in Supplementary Material; *P*s > 0.1) or negative mood (Figure S1C in Supplementary Material; *P*s > 0.05) measured by PANAS.

### Effect of Belief on Craving-Related Neural Activity

Next, we examined the effect of belief on craving-related neural activity (Figure 2B). Independent ROI analysis suggests that post-smoking SJWQ score was positively correlated with activity in the left mid insula (centered at [−38 2 4]) when smokers were told “nicotine in cigarette” and smoked a cigarette with nicotine (Figure 2B; Pearson’s *r* = 0.47, *P* < 0.05); such significant correlation was absent when smokers were told “no nicotine” and smoked a cigarette with nicotine (Figure 2B; Pearson’s *r* = −0.22, *P* > 0.1) and when smokers had a placebo cigarette (*P*s > 0.1). The differences between the correlation coefficients of craving-insula activation of told “nicotine” and smoked nicotine and all other conditions were also significant (*P*s < 0.05). Whole brain analysis also confirmed that the difference between craving-related left mid insula activations in the told “no nicotine” and smoked nicotine, and told “nicotine” and smoked nicotine conditions was significant (Figure 2B lower right; *P* < 0.05 corrected). This result suggests that belief about nicotine modulated craving-related insula activity when nicotine was present. In the absence of nicotine, however, such modulation effect of belief was not observed.

### Effect of Belief on Neural Activity Related to the Value Signal *r_**t**_*

**Figure 3:**
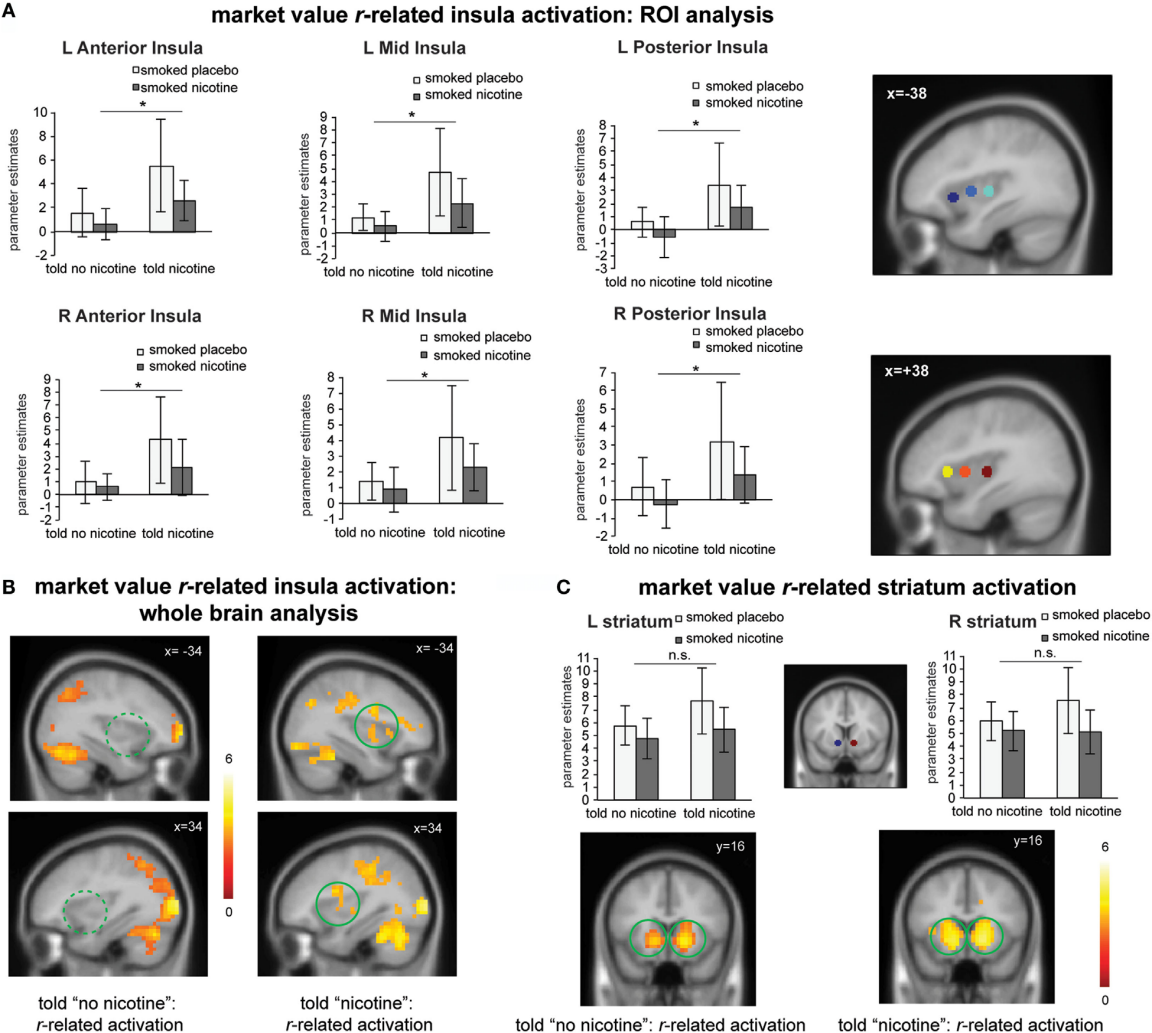
**(A)** Region-of-interest (ROI) analysis suggests that “told nicotine” elicited greater market value *r*-related activations in bilateral anterior, mid, and posterior insula regions compared to “told no nicotine” (main effect of belief, *P*s < 0.05). ROIs were selected from a meta-analysis on interoception from www.neurosynth.org. **(B)** Whole brain analysis confirmed that market value *r*-related insula activation was only significant when subjects were told “nicotine,” but not when they were told “no nicotine” (*P* < 0.05 corrected for family wise error). The contrast between the two maps is also significant for the insula (see Results, *P* < 0.05 corrected). **(C)** Both ROI and whole brain analysis show that striatum activation related to market value *r* did not significant differ between the “told nicotine” and “told no nicotine” conditions at the same threshold of **(A,B)**. The contrast between two conditions is also non-significant for the striatum even with small volume correction. *P ≤ 0.05. L, left; R, right. Error bar represents 95% confidence interval.

Next, we examined the effects of belief states on neural activity related to the value signal *r_t_* (see Table S1 in Supplementary Material for effect of belief on learning behavior). Independent ROI analysis suggests that for all insula ROIs (bilateral anterior, mid, and posterior insula), there was a significant main effect of belief [Figure 3A; *F*_(1,23)_ > 4.4, *P*s < 0.05]; there was no significant main effect of nicotine or belief–nicotine interaction (*F*s < 3, *P*s > 0.1). Whole brain analysis on *r_t_*-related activation also confirmed that bilateral insula regions were only activated when participants were told “nicotine in cigarette” but not when they were told “no nicotine” (Figure 3B; *P_FWE_* < 0.05).

For the striatum ROIs, there was no significant main effect of belief or nicotine, or their interaction (Figure 3C; *F*s < 4, *P*s > 0.05). Whole brain analysis suggests that *r_t_*-related striatum activation was equivalent for both the told “nicotine” and told “no nicotine” conditions (Figure 3C; *P*_FWE_ < 0.05). Taken together, these results suggest that value signals encoded in the insular cortex, but not in the ventral striatum, were modulated by belief about nicotine.

### Effect of Belief on Neural Activity Related to Both Craving and Learning Signals

**Figure 4:**
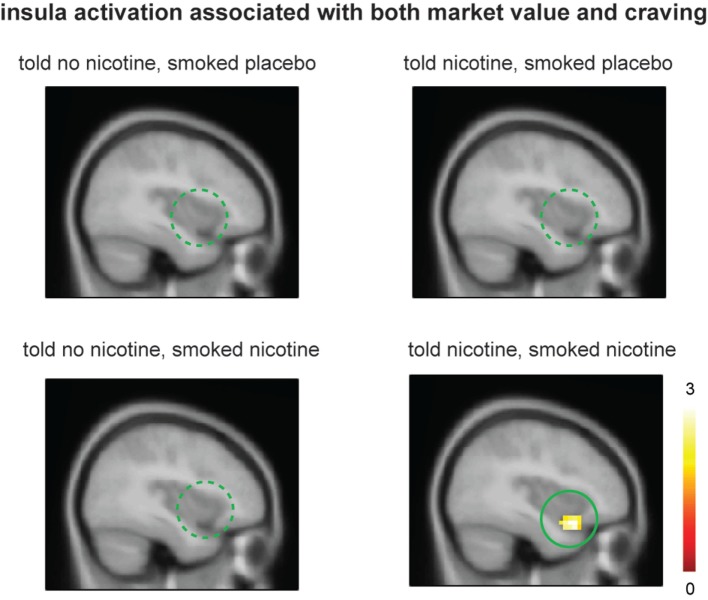
**Smokers showed significant ventral anterior insula activation related to both market value *r* and post-smoking craving only when they were told “nicotine in cigarette” and smoked nicotine but not in other conditions**. Activation maps were displayed at *P* < 0.05 for visualization purpose.

Lastly, we examined the effect of belief on brain regions that might encode both craving and reinforcement learning by correlating value-related activations with post-smoking craving at the group level. When smokers were “told nicotine” and smoked nicotine, there was significant ventral anterior insular activation that correlated to both craving and learning signals (Figure 4, lower right panel; *P* < 0.05 corrected); such activation was attenuated when smokers were told “no nicotine” (Figure 4, upper and lower left panels; *P* < 0.05 corrected). These results suggest that (1) the left ventral anterior insula integrates both subjective craving and decision-making signals and (2) such activity is dependent on belief about nicotine rather than nicotine in deprived smokers.

## Discussion

Our findings in deprived smokers are threefold. First, smoking a nicotine cigarette reduced craving only when subjects were told “nicotine in cigarette” but not otherwise. Second, craving-related insula activity was also modulated by belief about nicotine when subjects smoked a nicotine cigarette. Third, beliefs modulated insula activations related to computational learning signals (market value *r*) regardless of the presence of nicotine. These results provide further evidence top-down beliefs, in deprived smokers, can influence a powerful neuroactive substance like nicotine at both behavioral and neural levels.

Previous work has addressed addiction as a problem of aberrant dopamine function within the framework of reinforcement learning (5, 25, 52, 53). Nicotine, a nicotinic acetylcholine receptor agonist, is known to modulate dopamine neurotransmission (54, 55), which encodes reinforcement learning signals, such as reward and reward prediction errors (52, 56, 57). It is therefore not surprising that addicted individuals show aberrant dopamine-dependent reward processing (5, 43). Our previous work also shows that belief about the nicotine content in a cigarette modulates value and reward prediction error signals at both neural and behavioral levels in non-deprived smokers (15). Specifically, these results demonstrate that belief (expectancy) has a powerful effect in overriding the effects of nicotine on learning behavior and related neural activities in the striatum. These findings have far-reaching implications because (1) aberrant learning and decision making related to drug intake have been considered a hallmark of drug addiction and need to be “fixed”, and (2) learning is crucial for many aspects of cognition and leads to behavioral changes (53, 58, 59). Thus, by changing learning, we could potentially influence many important aspects of behavior and cognition.

One important question that remained unanswered was whether belief could also modulate drug-induced subjective states such as craving and associated patterns of brain activation. Craving is a core symptom of drug addiction. Its heterogeneity is caused by not only the substance or the belief but also different psychological effects like anxiety, depression or compulsive behavior (60). Here, we address this question and extend our previous finding by showing that belief has a significant impact on subjective craving and insula activity related to both craving and learning when participants smoked a cigarette with nicotine. We also show that the effect of belief on craving is not attributable to general changes in positive or negative mood. These results suggest that craving, a core symptom and an important “feeling” component of addiction, can also be modulated by top-down beliefs. This finding provides evidence supporting cognitive therapies targeting maladaptive beliefs in addiction (61) and cognitive strategies such as cognitive reappraisal and mindfulness, which attempt to attenuate craving by reinterpreting the craving signal (3, 62, 63). Moreover, the current study shows that neither belief nor nicotine alone reduces craving in deprived smokers, and that reduced craving was only observed when smokers were both told (cognitive belief) nicotine was present and nicotine was actually administered. This result offers a potential explanation for the unsatisfying outcome of pharmacological treatment (e.g., nicotine replacement therapy) and cognitive therapy when administered alone (4, 64, 65).

Several previous laboratory studies have also demonstrated the impact of cognitive factors on craving and related neural activities. Brody et al. reported increased anterior and posterior cingulate activations, and decreased activities in the cuneus, lateral occipital gyrus, and postcentral gyrus, when smokers actively tried to resist craving (1). Kober and colleagues asked smokers to think about short-term (pleasant) or long-term (harmful) consequences of smoking; such cognitive strategies significantly reduced craving (62), and increased neural activities in dorsomedial, dorsolateral, and ventrolateral prefrontal cortices, and decreased neural activities in ventral striatum, subgenual cingulate, amygdala, and ventral tegmental area (66). Using positron emission tomography (PET), Volkow and colleagues reported that cognitive strategy also inhibited craving and decreased brain metabolism in nucleus accumbens and orbitofrontal cortex in cocaine addicts (3). Moreover, Tang and colleagues showed that 2 weeks of mindfulness training produced a significant reduction in craving, accompanied by increased resting-state activity in anterior cingulate and prefrontal cortex in smokers (63). In contrast, our experimental manipulation only involved a simple suggestion of “nicotine in cigarette” or “no nicotine in cigarette,” rather than explicitly asking the participants to reduce craving using effortful control. This implies that belief could potentially be modified in a faster and easier fashion. Together with previous findings, the current results further demonstrate the influence of drug expectancies and beliefs in overriding the effect of nicotine on brain, behavior, and subjective states such as craving.

We also found that belief modulated activity in the insula related to both craving and learning signals when deprived smokers performed a sequential decision-making task. The insula is traditionally considered to process interoceptive information (67, 68), which provides the basis of emotional feelings (69–71). Heightened awareness and attention paid to bodily signals are also associated with increased insula activation (69, 72). Recently, an increasing number of studies have suggested that the insula is also a key neural region involved in complex cognition and decision making (26, 32, 44, 46, 72, 73), possibly by providing information of an agent’s internal states to exteroceptive decision-making processes (29). At the neuroanatomical level, the insula is known to contain high density of both nicotinic cholinergic receptors (74) as well as dopamine D1 receptors (75). The insula also receives strong dopaminergic innervations (76), and the disruption of these innervations directly disrupts aversive learning (77). It is therefore not surprising that malfunctioning of the insula has been associated with pathological decision making (2, 78) as well as subjective craving in addiction (1, 3). Attenuated insular cerebral blood flow has been observed in alcoholic subjects (79). In addition, insula–amygdala functional connectivity is elevated during nicotine withdrawal in cigarette smokers; such hyper-connectivity could be down regulated by varenicline and nicotine, two nicotinic acetylcholine receptor agonists and cessation aids for nicotine addiction (80). Damage to the insula disrupts craving and interoceptive representation of drugs in addiction (19, 81).

It is worth noting that belief selectively modulated subjective craving and insula activation, but not learning-related behavior (Table S1 in Supplementary Material) or striatum activity (Figure 3C) in deprived smokers. This is in sharp contrast to our previous finding of the impact of belief on striatal signals and choice behavior in non-deprived smokers (15). This finding suggests that what belief really had an impact on, and what the insula encoded essentially, was interoceptive information (i.e., craving) during decision making in deprived smokers, despite of the fact that insula activation covaried with both craving and learning signals when subjects were told “nicotine in cigarette” and smoked a nicotine cigarette (and not otherwise). Together with our previous finding in non-deprived smokers, these results also indicate that interoceptive states (i.e., deprived vs. non-deprived) could greatly shift the influence of beliefs to difference aspects of addiction (i.e., craving vs. reinforcement learning). We speculate that the insula could be a neural substrate integrating interoceptive and reinforcement learning aspects of addiction (25, 82) through: (1) the initial interoceptive state of the agent (“interoceptive representation”); (2) interoceptive value associated with drugs or drug cues (“interoceptive valuation”), (3) interoceptive consequences following choosing a drug/drug-cue vs. a non-drug stimulus (“interoceptive outcome evaluation”), and (4) updating the previous interoceptive state and valuation based on the interoceptive outcomes (“interoceptive learning”).

In summary, we show that in deprived smokers, belief about nicotine modulates subjective craving and activity in neural structures that process interoceptive information such as the insular cortex. These results provide compelling evidence supporting a strong influence of beliefs to counter drug effects on craving and addiction, as well as insights into the mechanisms of cognitive treatments for addiction.

## Author Contributions

XG analyzed data and wrote paper. TL designed experiment, analyzed data, and wrote paper. RS and PB collected data. AS designed experiment and wrote paper. UK analyzed data. PC designed experiment and wrote paper. PRM designed experiment, analyzed data, and wrote paper.

## Conflict of Interest Statement

The authors declare that the research was conducted in the absence of any commercial or financial relationships that could be construed as a potential conflict of interest.

## Footnotes

^1^http://www.covita.net/

^2^http://www.fil.ion.ucl.ac.uk/spm/software/spm8/

^3^www.neurosynth.org

## References

[1] Brody AL Mandelkern MA Olmstead RE Jou J Tiongson E Allen V Neural substrates of resisting craving during cigarette cue exposure. Biol Psychiatry (2007) 62:642–51. 10.1016/j.biopsych.2006.10.026 17217932 PMC1992815

[2] Goldstein RZ Craig AD Bechara A Garavan H Childress AR Paulus MP The neurocircuitry of impaired insight in drug addiction. Trends Cogn Sci (2009) 13:372–80. 10.1016/j.tics.2009.06.004 19716751 PMC2844118

[3] Volkow ND Fowler JS Wang GJ Telang F Logan J Jayne M Cognitive control of drug craving inhibits brain reward regions in cocaine abusers. Neuroimage (2010) 49:2536–43. 10.1016/j.neuroimage.2009.10.088 19913102 PMC2818484

[4] Nestler EJ. From neurobiology to treatment: progress against addiction. Nat Neurosci (2002) 5(Suppl):1076–9. 10.1038/nn945 12403990

[5] Dani JA Balfour DJ. Historical and current perspective on tobacco use and nicotine addiction. Trends Neurosci (2011) 34:383–92. 10.1016/j.tins.2011.05.001 21696833 PMC3193858

[6] De Biasi M Dani JA. Reward, addiction, withdrawal to nicotine. Annu Rev Neurosci (2011) 34:105–30. 10.1146/annurev-neuro-061010-113734 21438686 PMC3137256

[7] Polosa R Benowitz NL. Treatment of nicotine addiction: present therapeutic options and pipeline developments. Trends Pharmacol Sci (2011) 32:281–9. 10.1016/j.tips.2010.12.008 21256603 PMC5564372

[8] Robinson TE Berridge KC. The psychology and neurobiology of addiction: an incentive-sensitization view. Addiction (2000) 95(Suppl 2):S91–117. 10.1080/09652140050111681 11002906

[9] Volkow ND Wang GJ Fowler JS Tomasi D Telang F. Addiction: beyond dopamine reward circuitry. Proc Natl Acad Sci U S A (2011) 108:15037–42. 10.1073/pnas.1010654108 21402948 PMC3174598

[10] Gundersen H Specht K Gruner R Ersland L Hugdahl K. Separating the effects of alcohol and expectancy on brain activation: an fMRI working memory study. Neuroimage (2008) 42:1587–96. 10.1016/j.neuroimage.2008.05.037 18588989

[11] Yoder KK Morris ED Constantinescu CC Cheng TE Normandin MD O’connor SJ When what you see isn’t what you get: alcohol cues, alcohol administration, prediction error, and human striatal dopamine. Alcohol Clin Exp Res (2009) 33:139–49. 10.1111/j.1530-0277.2008.00821.x 18976347 PMC2905874

[12] Van Holst RJ Clark L Veltman DJ Van Den Brink W Goudriaan AE. Enhanced striatal responses during expectancy coding in alcohol dependence. Drug Alcohol Depend (2014) 142:204–8. 10.1016/j.drugalcdep.2014.06.019 25012896

[13] McBride D Barrett SP Kelly JT Aw A Dagher A. Effects of expectancy and abstinence on the neural response to smoking cues in cigarette smokers: an fMRI study. Neuropsychopharmacology (2006) 31:2728–38. 10.1038/sj.npp.1301075 16598192

[14] Robinson JD Engelmann JM Cui Y Versace F Waters AJ Gilbert DG The effects of nicotine dose expectancy and motivationally relevant distracters on vigilance. Psychol Addict Behav (2014) 28:752–60. 10.1037/a0035122 24841184 PMC4518546

[15] Gu X Lohrenz T Salas R Baldwin PR Soltani A Kirk U Belief about nicotine selectively modulates value and reward prediction error signals in smokers. Proc Natl Acad Sci U S A (2015) 112(8):2539–44. 10.1073/pnas.1416639112 25605923 PMC4345562

[16] Volkow ND Wang GJ Ma Y Fowler JS Zhu W Maynard L Expectation enhances the regional brain metabolic and the reinforcing effects of stimulants in cocaine abusers. J Neurosci (2003) 23:11461–8. 14673011 10.1523/JNEUROSCI.23-36-11461.2003 PMC6740524

[17] Kufahl P Li Z Risinger R Rainey C Piacentine L Wu G Expectation modulates human brain responses to acute cocaine: a functional magnetic resonance imaging study. Biol Psychiatry (2008) 63:222–30. 10.1016/j.biopsych.2007.03.021 17644071

[18] Paulus MP Tapert SF Schuckit MA. Neural activation patterns of methamphetamine-dependent subjects during decision making predict relapse. Arch Gen Psychiatry (2005) 62:761–8. 10.1001/archpsyc.62.7.761 15997017

[19] Naqvi NH Rudrauf D Damasio H Bechara A. Damage to the insula disrupts addiction to cigarette smoking. Science (2007) 315:531–4. 10.1126/science.1135926 17255515 PMC3698854

[20] Garavan H. Insula and drug cravings. Brain Struct Funct (2010) 214:593–601. 10.1007/s00429-010-0259-8 20512373

[21] Viswanath H Velasquez KM Savjani R Molfese DL Curtis K Molfese PJ Interhemispheric insular and inferior frontal connectivity are associated with substance abuse in a psychiatric population. Neuropharmacology (2015) 92:63–8. 10.1016/j.neuropharm.2014.12.030 25592214

[22] Contreras M Ceric F Torrealba F. Inactivation of the interoceptive insula disrupts drug craving and malaise induced by lithium. Science (2007) 318:655–8. 10.1126/science.1145590 17962567

[23] Forget B Pushparaj A Le Foll B. Granular insular cortex inactivation as a novel therapeutic strategy for nicotine addiction. Biol Psychiatry (2010) 68:265–71. 10.1016/j.biopsych.2010.01.029 20299008

[24] Versace F Engelmann JM Jackson EF Costa VD Robinson JD Lam CY Do brain responses to emotional images and cigarette cues differ? An fMRI study in smokers. Eur J Neurosci (2011) 34:2054–63. 10.1111/j.1460-9568.2011.07915.x 22097928 PMC3237919

[25] Everitt BJ Robbins TW. Neural systems of reinforcement for drug addiction: from actions to habits to compulsion. Nat Neurosci (2005) 8:1481–9. 10.1038/nn1579 16251991

[26] Naqvi NH Bechara A. The insula and drug addiction: an interoceptive view of pleasure, urges, and decision-making. Brain Struct Funct (2010) 214:435–50. 10.1007/s00429-010-0268-7 20512364 PMC3698865

[27] Critchley HD Harrison NA. Visceral influences on brain and behavior. Neuron (2013) 77:624–38. 10.1016/j.neuron.2013.02.008 23439117

[28] Seth AK. Interoceptive inference, emotion, and the embodied self. Trends Cogn Sci (2013) 17:565–73. 10.1016/j.tics.2013.09.007 24126130

[29] Gu X Fitzgerald TH Interoceptive inference: homeostasis and decision-making. Trends Cogn Sci (2014) 18:269–70. 10.1016/j.tics.2014.02.001 24582825

[30] Seymour B O’doherty JP Dayan P Koltzenburg M Jones AK Dolan RJ Temporal difference models describe higher-order learning in humans. Nature (2004) 429:664–7. 10.1038/nature02581 15190354

[31] Preuschoff K Quartz SR Bossaerts P. Human insula activation reflects risk prediction errors as well as risk. J Neurosci (2008) 28:2745–52. 10.1523/JNEUROSCI.4286-07.2008 18337404 PMC6670675

[32] Xiang T Lohrenz T Montague PR. Computational substrates of norms and their violations during social exchange. J Neurosci (2013) 33:1099a–108a. 10.1523/JNEUROSCI.1642-12.2013 23325247 PMC3631781

[33] Simmons WK Rapuano KM Kallman SJ Ingeholm JE Miller B Gotts SJ Category-specific integration of homeostatic signals in caudal but not rostral human insula. Nat Neurosci (2013) 16:1551–2. 10.1038/nn.3535 24077565 PMC3835665

[34] Michael RB Garry M Kirsch I Suggestion, cognition, and behavior. Curr Dir Psychol Sci (2012) 21:151–6. 10.1177/0963721412446369

[35] Oakley DA Halligan PW. Hypnotic suggestion: opportunities for cognitive neuroscience. Nat Rev Neurosci (2013) 14:565–76. 10.1038/nrn3538 23860312

[36] American Psychiatric Association. Diagnostic and Statistical. Manual of Mental Disorders, 4th ed DSM-IV-TR^®^ Arlington, VA: American Psychiatric Association (2000).

[37] Falk DE Yi H Hiller-Sturmhofel S An epidemiologic analysis of co-occurring alcohol and tobacco use and disorders. Alcohol Res Health (2006) 29:162–71. 17373404 PMC6527037

[38] Harrell PT Juliano LM. A direct test of the influence of nicotine response expectancies on the subjective and cognitive effects of smoking. Exp Clin Psychopharmacol (2012) 20:278–86. 10.1037/a0028652 22708609 PMC6413876

[39] Macqueen DA Heckman BW Blank MD Janse Van Rensburg K Evans DE Drobes DJ. Transient compensatory smoking in response to placebo cigarettes. Psychopharmacology (Berl) (2012) 223:47–54. 10.1007/s00213-012-2685-1 22427021 PMC3802524

[40] Shiffman SM Jarvik ME Smoking withdrawal symptoms in two weeks of abstinence. Psychopharmacology (Berl) (1976) 50:35–9. 10.1007/BF00634151 827760

[41] Watson D Clark LA Tellegen A. Development and validation of brief measures of positive and negative affect: the PANAS scales. J Pers Soc Psychol (1988) 54:1063–70. 10.1037/0022-3514.54.6.1063 3397865

[42] Lohrenz T McCabe K Camerer CF Montague PR. Neural signature of fictive learning signals in a sequential investment task. Proc Natl Acad Sci U S A (2007) 104:9493–8. 10.1073/pnas.0608842104 17519340 PMC1876162

[43] Chiu PH Lohrenz TM Montague PR. Smokers’ brains compute, but ignore, a fictive error signal in a sequential investment task. Nat Neurosci (2008) 11:514–20. 10.1038/nn2067 18311134

[44] Gu X Kirk U Lohrenz TM Montague PR. Cognitive strategies regulate fictive, but not reward prediction error signals in a sequential investment task. Hum Brain Mapp (2014) 35:3738–49. 10.1002/hbm.22433 24382784 PMC4105325

[45] Faraway JJ Extending the Linear Model with R: Generalized Linear, Mixed Effects and Nonparametric Regression Models. Boca Raton, FL: Taylor & Francis (2005).

[46] Gu X Wang X Hula A Wang S Xu S Lohrenz TM Necessary, yet dissociable contributions of the insular and ventromedial prefrontal cortices to norm adaptation: computational and lesion evidence in humans. J Neurosci (2015) 35:467–73. 10.1523/JNEUROSCI.2906-14.2015 25589742 PMC4293403

[47] Team RC R: A Language and Environment for Statistical Computing. Vienna, Austria: R Foundation for Statistical Computing (2011) Available from: http://www.R-project.org/

[48] Pinheiro J Bates D Debroy S Sarkar D R Development Core Team nlme: Linear and Nonlinear Mixed Effects Models. R Package Version 3.1-113. (2013).

[49] Warnes GR Bolker B Lumley T Johnson RC gmodels: Various R Programming Tools for Model Fitting. R Package Version 2.15.4.1. (2013). Available from: http://CRAN.R-project.org/package=gmodels

[50] Slotnick SD Moo LR Segal JB Hart J Jr. Distinct prefrontal cortex activity associated with item memory and source memory for visual shapes. Brain Res Cogn Brain Res (2003) 17:75–82. 10.1016/S0926-6410(03)00082-X 12763194

[51] Gu X Eilam-Stock T Zhou T Anagnostou E Kolevzon A Soorya L Autonomic and brain responses associated with empathy deficits in autism spectrum disorder. Hum Brain Mapp (2015) 36:3323–38. 10.1002/hbm.22840 25995134 PMC4545680

[52] Montague PR Hyman SE Cohen JD. Computational roles for dopamine in behavioural control. Nature (2004) 431:760–7. 10.1038/nature03015 15483596

[53] Redish AD. Addiction as a computational process gone awry. Science (2004) 306:1944–7. 10.1126/science.1102384 15591205

[54] Zhou FM Liang Y Dani JA. Endogenous nicotinic cholinergic activity regulates dopamine release in the striatum. Nat Neurosci (2001) 4:1224–9. 10.1038/nn769 11713470

[55] Rice ME Cragg SJ. Nicotine amplifies reward-related dopamine signals in striatum. Nat Neurosci (2004) 7:583–4. 10.1038/nn1244 15146188

[56] Schultz W Dayan P Montague PR. A neural substrate of prediction and reward. Science (1997) 275:1593–9. 10.1126/science.275.5306.1593 9054347

[57] Montague PR King-Casas B Cohen JD. Imaging valuation models in human choice. Annu Rev Neurosci (2006) 29:417–48. 10.1146/annurev.neuro.29.051605.112903 16776592

[58] Rangel A Camerer C Montague PR. A framework for studying the neurobiology of value-based decision making. Nat Rev Neurosci (2008) 9:545–56. 10.1038/nrn2357 18545266 PMC4332708

[59] Volkow ND Baler R Beliefs modulate the effects of drugs on the human brain. Proc Natl Acad Sci U S A (2015) 112:2301–2. 10.1073/pnas.1500552112 25673784 PMC4345612

[60] Schlaff G Walter H Lesch OM. The Lesch alcoholism typology – psychiatric and psychosocial treatment approaches. Ann Gastroenterol (2011) 24:89–97. 24713718 PMC3959295

[61] Beck AT Wright FD Newman CF Liese BS Cognitive Therapy of Substance Abuse. New York: Guilford Press (2011). 8289917

[62] Kober H Kross EF Mischel W Hart CL Ochsner KN Regulation of craving by cognitive strategies in cigarette smokers. Drug Alcohol Depend (2010) 106:52–5. 10.1016/j.drugalcdep.2009.07.017 19748191 PMC2814914

[63] Tang YY Tang R Posner MI. Brief meditation training induces smoking reduction. Proc Natl Acad Sci U S A (2013) 110:13971–5. 10.1073/pnas.1311887110 23918376 PMC3752264

[64] Carroll KM Onken LS. Behavioral therapies for drug abuse. Am J Psychiatry (2005) 162:1452–60. 10.1176/appi.ajp.162.8.1452 16055766 PMC3633201

[65] Cahill K Stevens S Perera R Lancaster T. Pharmacological interventions for smoking cessation: an overview and network meta-analysis. Cochrane Database Syst Rev (2013) 5:CD009329. 10.1002/14651858.CD009329.pub2 23728690 PMC8406789

[66] Kober H Mende-Siedlecki P Kross EF Weber J Mischel W Hart CL Prefrontal-striatal pathway underlies cognitive regulation of craving. Proc Natl Acad Sci U S A (2010) 107:14811–6. 10.1073/pnas.1007779107 20679212 PMC2930456

[67] Penfield W Faulk ME Jr The insula; further observations on its function. Brain (1955) 78:445–70. 10.1093/brain/78.4.445 13293263

[68] Phillips ML Young AW Senior C Brammer M Andrew C Calder AJ A specific neural substrate for perceiving facial expressions of disgust. Nature (1997) 389:495–8. 10.1038/39051 9333238

[69] Critchley HD Wiens S Rotshtein P Ohman A Dolan RJ. Neural systems supporting interoceptive awareness. Nat Neurosci (2004) 7:189–95. 10.1038/nn1176 14730305

[70] Craig AD How do you feel – now? The anterior insula and human awareness. Nat Rev Neurosci (2009) 10:59–70. 10.1038/nrn2555 19096369

[71] Gu X Hof PR Friston KJ Fan J. Anterior insular cortex and emotional awareness. J Comp Neurol (2013) 521:3371–88. 10.1002/cne.23368 23749500 PMC3999437

[72] Kirk U Gu X Harvey AH Fonagy P Montague PR. Mindfulness training modulates value signals in ventromedial prefrontal cortex through input from insular cortex. Neuroimage (2014) 100:254–62. 10.1016/j.neuroimage.2014.06.035 24956066 PMC4140407

[73] Bossaerts P. Risk and risk prediction error signals in anterior insula. Brain Struct Funct (2010) 214:645–53. 10.1007/s00429-010-0253-1 20512378

[74] Picard F Sadaghiani S Leroy C Courvoisier DS Maroy R Bottlaender M. High density of nicotinic receptors in the cingulo-insular network. Neuroimage (2013) 79:42–51. 10.1016/j.neuroimage.2013.04.074 23631995

[75] Hurd YL Suzuki M Sedvall GC. D1 and D2 dopamine receptor mRNA expression in whole hemisphere sections of the human brain. J Chem Neuroanat (2001) 22:127–37. 10.1016/S0891-0618(01)00122-3 11470560

[76] Gaspar P Berger B Febvret A Vigny A Henry JP. Catecholamine innervation of the human cerebral cortex as revealed by comparative immunohistochemistry of tyrosine hydroxylase and dopamine-beta-hydroxylase. J Comp Neurol (1989) 279:249–71. 10.1002/cne.902790208 2563268

[77] Zito KA Bechara A Greenwood C Van Der Kooy D. The dopamine innervation of the visceral cortex mediates the aversive effects of opiates. Pharmacol Biochem Behav (1988) 30:693–9. 10.1016/0091-3057(88)90086-X 3211979

[78] Paulus MP Decision-making dysfunctions in psychiatry – altered homeostatic processing? Science (2007) 318:602–6. 10.1126/science.1142997 17962553

[79] Sullivan EV Muller-Oehring E Pitel AL Chanraud S Shankaranarayanan A Alsop DC A selective insular perfusion deficit contributes to compromised salience network connectivity in recovering alcoholic men. Biol Psychiatry (2013) 74:547–55. 10.1016/j.biopsych.2013.02.026 23587427 PMC3766441

[80] Sutherland MT Carroll AJ Salmeron BJ Ross TJ Hong LE Stein EA. Down-regulation of amygdala and insula functional circuits by varenicline and nicotine in abstinent cigarette smokers. Biol Psychiatry (2013) 74:538–46. 10.1016/j.biopsych.2013.01.035 23506999 PMC3775982

[81] Dani JA Montague PR Disrupting addiction through the loss of drug-associated internal states. Nat Neurosci (2007) 10:403–4. 10.1038/nn0407-403 17387327

[82] Naqvi NH Bechara A. The hidden island of addiction: the insula. Trends Neurosci (2009) 32:56–67. 10.1016/j.tins.2008.09.009 18986715 PMC3698860

