# Supplementary material for: Belief about Nicotine Modulates Subjective Craving and Insula Activity in Deprived Smokers
Source: Front Psychiatry. 2016 Jul 13;7:126. doi: 10.3389/fpsyt.2016.00126 (PMC4942468; doi:10.3389/fpsyt.2016.00126)
Supplement: Supplementary file 1 [file data_sheet_1.docx]

**Supplementary Material**

**Belief about nicotine modulates subjective craving and insula activity in deprived smokers**

**Xiaosi Gu^1,2^, Terry M. Lohrenz^3^, Ramiro Salas^4^, Philip R. Baldwin^4^, Alireza Soltani^5^, Ulrich Kirk^6^, Paul M. Cinciripini^7^, and P. Read Montague^1,3,8^**

1. Wellcome Trust Centre for Neuroimaging, University College London, London WC1N 3BG, United Kingdom

2. School of Behavioral and Brain Sciences, The University of Texas at Dallas, Dallas, TX 75235

3. Human Neuroimaging Laboratory, Virginia Tech Carilion Research Institute, Roanoke, VA 24011, United States

4. Menninger Department of Psychiatry and Behavioral Sciences, Baylor College of Medicine, Houston, Texas 77225, United States

5. Department of Psychological and Brain Sciences, Dartmouth College, 6207 Moore Hall, Hanover, NH 03755, United States

6. Institute of Psychology, University of Southern Denmark, Campusvej 55, DK-5230 Odense, Denmark

7. Departments of Behavioral Science, The University of Texas MD Anderson Cancer Center, Houston, TX 77230, United States

8. Department of Physics, Virginia Polytechnic Institute and State University, Blacksburg VA, United States

* Correspondence:

Xiaosi Gu, Ph.D

School of Behavioral and Brain Sciences, The University of Texas at Dallas, Dallas, TX 75235

Email: [xiaosi.gu@utdallas.edu](mailto:xiaosi.gu@utdallas.edu)

Tel: +1) 972-883-3303

Fax: +1) 214-905-3026

**Supplementary Figure 1. A**) The time lag between the two measurements was comparable between conditions. There was no significant main effect of belief or nicotine, or interaction effect on either **B)** positive or **C)** negative mood measured by the Positive and Negative Affect Schedule (PANAS).

**Supplementary Table 1.** Behavioral regression model examining the effect of market return *r_t_* on normalized next bet $\tilde{b}_{t+1}$*_._* told0: told “no nicotine”; told1: told “nicotine”; nic0: cigarettes without nicotine; nic1: cigarettes with nicotine.

| **Item** | **Coefficient** | **Standard Error** | **t value** | ***P* value** |
| --- | --- | --- | --- | --- |
| **Regressor** |  |  |  |  |
| told0 nic0 | -0.038 | 0.012 | -3.089 | 0.0020 |
| told1 nic0 | -0.033 | 0.012 | -2.699 | 0.0070 |
| told0 nic1 | -0.039 | 0.012 | -3.170 | 0.0015 |
| told1 nic1 | -0.036 | 0.013 | -2.847 | 0.0044 |
| told0 nic0: $\tilde{b}_{t}$ | 0.518 | 0.050 | 10.326 | <0.0001 |
| told1 nic0: $\tilde{b}_{t}$ | 0.503 | 0.050 | 10.037 | <0.0001 |
| told0 nic1: $\tilde{b}_{t}$ | 0.527 | 0.050 | 10.509 | <0.0001 |
| told1 nic1: $\tilde{b}_{t}$ | 0.499 | 0.050 | 9.950 | <0.0001 |
| told0 nic0: *r_t_* | 4.652 | 0.592 | 7.861 | <0.0001 |
| told1 nic0: *r_t_* | 4.425 | 0.591 | 7.493 | <0.0001 |
| told0 nic1: *r_t_* | 4.491 | 0.589 | 7.621 | <0.0001 |
| told1 nic1: *r_t_* | 4.461 | 0.596 | 7.480 | <0.0001 |
| **Linear contrasts** |  |  |  |  |
| (told1nic0- told0 nic0): *r_t_* | -0.226 | 0.266 | -0.852 | 0.394 |
| (told1nic1- told0 nic1): *r_t_* | -0.031 | 0.273 | -0.113 | 0.910 |
| nic1-nic0: *r_t_* | -0.062 | 0.189 | -0.330 | 0.741 |
